# T20. SEARCHING FOR NOVEL AUTOANTIBODIES WITH CLINICAL RELEVANCE IN PSYCHIATRIC DISORDERS

**DOI:** 10.1093/schbul/sby016.296

**Published:** 2018-04-01

**Authors:** Mats Persson, Arasch Zandian, Louise Wingård, Hanna Nilsson, Evelina Sjöstedt, Daniel Johansson, David Just, Cecilia Hellström, Mathias Uhlén, Jochen Schwenk, Anna Häggmark-Månberg, Oscar Norbeck, Björn Owe-Larsson, Peter Nilsson

**Affiliations:** 1 Karolinska Institutet; 2 Royal Institute of Technology; 3 Uppsala University

## Abstract

**Background:**

Immunological reactions may have a role in subgroups of patients suffering from psychiatric disorders. Possible markers for such subgroups may be autoantibodies of currently unknown nature. If identified, they could indicate which patients that would benefit from immunomodulatory treatment in addition to standard interventions. Modern proteomic methods allow analyses of antibody binding to thousands of different human proteins, facilitating the identification of currently undiscovered autoantibodies.

**Methods:**

We have explored the association between any seroreactivity in plasma samples from first episode psychosis patients against more than 2000 randomly chosen protein fragments derived from human proteins, and the development of disorders characterized by chronic or relapsing psychotic symptoms. Plasma from 53 patients and 41 non-psychotic controls were assessed; the clinical course of the patients were followed for a mean duration of 7 years. The plasma samples were analyzed for IgG reactivity to 2304 fragments (approx 100 a.a. residues in length) of human proteins using a multiplexed afﬁnity proteomic technique, and positive hits validated for binding in two additional assays.

**Results:**

Thirty patients were diagnosed with schizophrenia, delusional disorder, schizoaffective disorder, bipolar disorder or a long-term unspeciﬁed nonorganic psychosis during follow-up, while 23 patients achieved complete remission. Eight patient samples showed autoreactivity to the N-terminal fragment of the PAGE protein family (PAGE2B/PAGE2/PAGE5), whereas no such autoreactivity was seen among the controls. PAGE autoreactivity was associated with a signiﬁcantly increased risk of being diagnosed with schizophrenia during follow-up (odds ratio 6.7). An antisera raised against the N-terminal fragment stained an unknown extracellular target in human cortical brain tissue (Zandian et al., Transl Psychiatry 7: e1177; doi:10.1038/tp.2017.160).

We are currently investigating the identity of this target. In addition, two other putative new autoantibodies found primarily among the patients, and rarely in the controls, will be discussed at the meeting.

**Discussion:**

Our ﬁndings suggest that autoreactivity to the N-terminal portion of the PAGE protein family is associated with schizophrenia in a subset of patients with ﬁrst-episode psychosis. In addition, we propose that searching for novel autoantibodies in an unbiased way may be feasible using state-of-the-art proteomic methods, and can yield useful biological markers for immune involvement in subgroups of individuals diagnosed with psychiatric disorders.

